# Application of an optimized flow cytometry-based quantification of Platelet Activation (PACT): Monitoring platelet activation in platelet concentrates

**DOI:** 10.1371/journal.pone.0172265

**Published:** 2017-02-16

**Authors:** Cécile H. Kicken, Mark Roest, Yvonne M. C. Henskens, Bas de Laat, Dana Huskens

**Affiliations:** 1 Department of Anesthesiology and Pain Therapy, Maastricht University Medical Center, Maastricht, the Netherlands; 2 Synapse Research Institute, Maastricht, the Netherlands; 3 Department of Biochemistry, Cardiovascular Research Institute, Maastricht University, Maastricht, the Netherlands; 4 Central Diagnostic Laboratory, Maastricht University Medical Center, Maastricht, the Netherlands; Queen Mary University of London, UNITED KINGDOM

## Abstract

**Background:**

Previous studies have shown that flow cytometry is a reliable test to quantify platelet function in stored platelet concentrates (PC). It is thought that flow cytometry is laborious and hence expensive. We have optimized the flow cytometry-based quantification of agonist induced platelet activation (PACT) to a labor, time and more cost-efficient test. Currently the quality of PCs is only monitored by visual inspection, because available assays are unreliable or too laborious for use in a clinical transfusion laboratory. Therefore, the PACT was applied to monitor PC activation during storage.

**Study design and methods:**

The optimized PACT was used to monitor 5 PCs during 10 days of storage. In brief, optimized PACT uses a ready-to-use reaction mix, which is stable at -20°C. When needed, a test strip is thawed and platelet activation is initiated by mixing PC with PACT. PACT was based on the following agonists: adenosine diphosphate (ADP), collagen-related peptide (CRP) and thrombin receptor-activating peptide (TRAP-6). Platelet activation was measured as P-selectin expression. Light transmission aggregometry (LTA) was performed as a reference.

**Results:**

Both PACT and LTA showed platelet function decline during 10-day storage after stimulation with ADP and collagen/CRP; furthermore, PACT showed decreasing TRAP-induced activation. Major differences between the two tests are that PACT is able to measure the status of platelets in the absence of agonists, and it can differentiate between the number of activated platelets and the amount of activation, whereas LTA only measures aggregation in response to an agonist. Also, PACT is more time-efficient compared to LTA and allows high-throughput analysis.

**Conclusion:**

PACT is an optimized platelet function test that can be used to monitor the activation of PCs. PACT has the same accuracy as LTA with regard to monitoring PCs, but it is superior to both LTA and conventional flow cytometry based tests with regard to labor-, time- and cost efficiency.

## Introduction

The gold standard for testing platelet function is light transmission aggregometry (LTA) [1], which was described for the first time by Born and Cross in 1963 [2]. By adding a panel of agonists to platelet rich plasma (PRP), the platelets aggregate in an GPIIb/IIIa-dependent manner and an increase in light transmission through the plasma can be measured. This method has its limits. LTA is relatively insensitive to small changes in platelet function, it does not show pre-activation of the platelets and it may be affected by i.e. hemolysis or extremes in platelet count, it needs a relatively large sample volume, and the laborious procedure does not allow automated measurement or evaluation of a large number of samples simultaneously [3, 4]. Other fast tests available in clinical labs, such as Multiplate (multiple electrode impedance aggregometry), rotational thromboelastometry (ROTEM), thromboelastography (TEG), the platelet function analyzer (PFA)-100 or the verifyNOW, are all based on the formation of a platelet aggregate or blood clot. These methods are not as laborious as LTA, but are also unable to show pre-activation of platelets, do not allow high-throughput analysis and are insensitive in case of low platelet numbers [4]. Flow cytometry has been established for a long time as reliable technique for the quantification of platelet activation, also in thrombocytopenic patients. Despite the findings of several groups that flow cytometry in whole blood is superior to LTA with regard to performance, blood consumption and reproducibility [5], flow cytometry has not become a standard for platelet function testing in routine clinical settings. One of the reasons for this is that flow cytometry is commonly associated with complicated techniques and time consuming procedures, which have made the translation to a clinical setting unattractive.

Platelet concentrates (PCs) are used as a bleeding prophylaxis for patients suffering from a deep thrombocytopenia (platelet concentration < 10 x 10^9^/L, e.g. patients suffering from malignancies) or for patients with massive blood loss during surgery or trauma [6]. It is of utmost importance that the platelets in these concentrates are viable and form adequate aggregates to ensure hemostasis in PC transfusion recipients. This argues for an urgent need of reliable platelet function testing of these concentrates. Following current transfusion guidelines, platelet concentrates are only inspected visually for presence of the so-called swirl before issue to a patient [7, 8]. Swirl is a morphologic feature whose absence is associated with poor platelet viability [9]. However, swirl is not a direct and sensitive method to detect differences in platelet function and it lacks sensitivity and reproducibility. Platelet function testing seems necessary to guarantee the quality of PCs, but it is not a routine procedure at blood banks or at clinical transfusion labs, because of the time and labor intensity of currently available tests, and the lack of a test that is validated for post-transfusion outcome.

A useful method for testing PCs in a clinical setting would need to be sensitive for variation in platelet function, correlate with post-transfusion platelet function, be reproducible irrespective of operator experience, and be time and labor efficient [10]. Although we recently showed that LTA is capable of testing platelet function in PCs, it does not fit the desired characteristics of a useful test in a clinical setting [11, 12]. Additionally, there is little data to show that LTA can predict in vivo recovery of transfused platelets [13]. We have made major modifications to a flow cytometry based platelet activation test (PACT) to improve the time, labor and cost efficiency of the test.

The current study describes the evaluation of the quality of five PCs prepared from pooled buffy-coat platelets in plasma and stored up to 10 days using the optimized PACT, and compared it to LTA. Additionally, blood count and metabolic changes were studied to monitor storage conditions.

## Materials and methods

### Study design

The study met all institutional ethics requirements according to the Declaration of Helsinki (2015). At the local blood bank (Sanquin Blood Services, Amsterdam, the Netherlands), blood was drawn from healthy donors who met standard donation criteria and gave written, informed consent to use their blood for scientific purposes. PCs prepared from blood donated by consenting individuals were subsequently purchased from the blood bank. The current in vitro study is not subjected to the Dutch Medical Research Involving Human Subjects Act and therefore did not require approval of the local medical ethics board. Five fresh PCs were delivered at day 1 after blood donation (CompoFlex storage bag (Fresenius Kabi, Bad Homburg, Germany). These 5 PCs were tested as a pilot, it was deemed unethical to take more concentrates from patient care. Each concentrate consisted of randomly pooled leukoreduced buffy-coat derived platelets from five healthy donors, resuspended in plasma from one donor, with approximate volume 250–300 ml and platelet content 300–370·10^9^/PC. PC bags were stored at 22°C ± 2°C with continuous gentle agitation until testing. PCs were tested until day 10 after donation, to investigate PC function beyond both European and US storage guidelines (7 and 5 days, respectively) [7, 8]. From day 2 until day 10 after donation, 15 ml sample was collected once daily from each bag using a sterile 20 ml syringe, via a sterile spike with ultraport (B.Braun, Melsungen, Germany). The samples were processed immediately after collection. One part of the PC samples (± 10 ml) was used to prepare platelet poor plasma (PPP) by double centrifugation of the PCs at 2900 g for 10 min. PPP was used to prepare PRP with a platelet concentration of 200·10^9^/L.

### Reagents

For light transmission aggregometry (LTA), the following platelet agonists were purchased: the P2Y_1_/P2Y_12_ agonist ADP (Chrono-Log, Havertown, US), the glycoprotein VI (GPVI) agonist collagen (Chrono-Log, Havertown, USA, and the PAR-1 agonist thrombin receptor activator peptide-14 ((SFLLRNPNKYEPF), Bachem, Weil am Rhein, Germany). For our flow cytometric assay, ADP (01897, Sigma-Aldrich, Zwijndrecht, the Netherlands), TRAP-6 ((SFLLRN), Bachem, Weil am Rhein, Germany) and collagen-related peptide (CRP, Professor Farndale, university of Cambridge, United Kingdom) were purchased. Professor Farndale identified fragments of collagen, synthesized and assembled in active, triple-helical conformation and in this study, a single batch of cross-linked CRP (CRP-XL) was used. The antibodies used in this flow cytometry assay were PE-conjugated anti-P-selectin and APC-conjugated CD42b from BD-Biosciences (BD PharmingenTM, Franklin Lakes, USA) and FITC-conjugated anti-fibrinogen was purchased from DAKO (F0111, Dako, Glostrup, Denmark).

### Metabolic parameters

The pH and the glucose and lactate levels were measured immediately after sampling with a GEM Premier 4000 blood-gas analyzer (Instrumentation Laboratory, Zaventem, Belgium) at 37°C. As the upper measuring limit for lactate of this analyzer is 20 mmol/L, some samples were diluted 1:1 with 0.9% saline to accurately determine lactate levels. Platelet concentration and mean platelet volume (MPV) were determined in the citrated plasma with a Coulter cell counter (Beckman Coulter, Woerden, the Netherlands).

### Light transmission aggregometry

The gold standard light transmission aggregometry (LTA) was performed on a PAR-4 platelet aggregometer (Hart Biologicals, Hartlepool, United Kingdom) using unaltered PC. The light transmission at theoretical total aggregation was set using autologous PPP. Aggregation was measured in response to ADP, collagen, and TRAP-14, with final concentrations of 10 μmol/L, 4 μg/ml, and 30 μmol/L, respectively. A disposable microcuvette with stirring flea was filled with 200 μl PC and incubated at 37°C for 3 minutes. Subsequently, 20 μl agonist was added and aggregation was measured immediately for 10 minutes by amount of light transmitted through the cuvette. Aggregation was expressed as % maximum aggregation compared to PPP.

### Platelet activation test

The platelet activation test (PACT) was performed as described previously [14], using pre-prepared strips stored at -20°C containing a reaction mix and antibodies that is stable for at least 6 months [14]. PRP with a platelet concentration of 200·10^9^/L was further diluted by adding 50 μl PRP into 350 μl HEPES-buffered saline (HBS, 10 mmol/L HEPES, 150 mmol/L NaCl, 1 mmol/L MgSO_4_, 5 mmol/L KCL, pH 7.4). Of the diluted PC, 10 μl was added to 20 μl reaction mix consisting of agonists (ADP, CRP or TRAP-6), 87.5 μl/ml PE-conjugated anti-P-selectin, 87.5 μl/ml FITC-conjugated anti-fibrinogen and 12.5 μl/ml APC-conjugated anti-CD42b in HEPES-buffered saline. Reactions were stopped by adding 250 μl fixation solution (137 mmol/L NaCl, 2.7 mmol/L KCl, 1.12 mmol/L NaH_2_PO_4_, 1.15 mmol/L KH_2_PO_4_, 10.2 mmol/L Na_2_HPO_4_, 4 mmol/L EDTA, 0.5% formaldehyde) after 20 min of incubation at room temperature. Platelet activation was measured in dilution series of ADP (125, 31, 8, 2, 0.5, 0.1, 0.03 μmol/L), CRP (2500, 625, 156, 39, 10. 2, 0.6 ng/ml) and TRAP-6 (30, 10, 3, 1, 0.4, 0.4, 0.04 μmol/L) to find the optimal agonist concentrations for the PCs. Flow cytometry was used to distinguish between platelets and other cells on forward and sideward scatter pattern and by gating on the CD42b positive cells. Fluorescent intensity in the PE gate was selected to determine P-selectin density and fluorescent intensity in the FITC gate was used to determine fibrinogen binding, which indicates GPIIb/IIIa activation. Results are expressed as median fluorescent intensity (MFI). In the control samples, where no agonist was added, a marker was placed to indicate the non-activated cells. With this marker, the percentage of positive cells in the PCs was determined after adding different agonists.

### Statistics

The Statistical Package for the Social Sciences (SPSS) was used. The paired T-test was used to determine statistical significance within the PCs. A *P*-value <0.01 was considered statistically significant. Because of the small sample size n = 5, p-values alone are less likely to show a relevant change. Therefore, additionally the mean of day 2 (baseline) minus 2 standard deviations (mean -2SD) was used as a cut-off. Furthermore, simple linear regression analysis was performed to show trends. Figures were generated using Prism version 6 (GraphPad Software Inc., La Jolla, USA).

## Results

### Metabolic parameters

The pH and the lactate and glucose levels of all 5 PCs were measured from day 2 until day 10 after donation (Table 1). Platelets are metabolically active, which is reflected by a significant decrease of the glucose level in a linear fashion of 0.78 mmol/day (*r*^*2*^ = 0.997), accompanied by a linearly increasing lactate level of 1.25 mmol/day (*r*^*2*^ = 0.99). The pH also decreased in a linear fashion during storage, but never dropped below the critical level of 6.8 [15]. In addition, platelet concentration and mean platelet volume (MPV) were measured, however, no change in time was observed. Platelet concentration was only significantly lower at day 5 after donation; the MPV did not change significantly on any measured day (Table 1).

**Table 1:** Metabolic parameters of the platelet concentrates.

Day	pH	Glucose (mmol/L)	Lactate (mmol/L)	Platelet conc. (10^9^ cells/L)	Mean platelet volume (fl)
**2**	7.33±0.35	16.36±1.40	9.74±1.18	1008.20±52.71	7.64±0.16
**3**	7.28±0.39*	15.94±1.66	11.70±1.55*	1016.20±55.19	7.68±0.18
**4**	7.29±0.05	14.8±1.60**	12.82±1.54**	997.80±48.65	7.50±0.15
**5**	7.27±0.05*	14.30±1.54**	14.30±1.74**	976.80±54.45*	7.60±0.18
**6**	7.22±0.06*	13.50±1.70**	15.80±1.96**	990.20±50.32	7.54±0.20
**7**	7.22±0.06*	12.52±1.67**	16.82±1.47**	1001.00±55.26	7.66±0.20
**8**	7.17±0.10*	11.88±1.84**	17.72±1.50**	1000.2±54.49	7.68±0.20
**9**	7.14±0.09*	11.18±2.00**	19.16±1.39**	111.40±50.52	7.76±0.22
**10**	7.06±0.11*	10.20±2.22**	19.78±1.30**	1012.20±61.59	7.74±0.19

Data are expressed as mean±SD.

* = P<0.01 and

** = P<0.001 compared to baseline (day 2 after donation).

### Platelet aggregation tests

**Fig 1:**
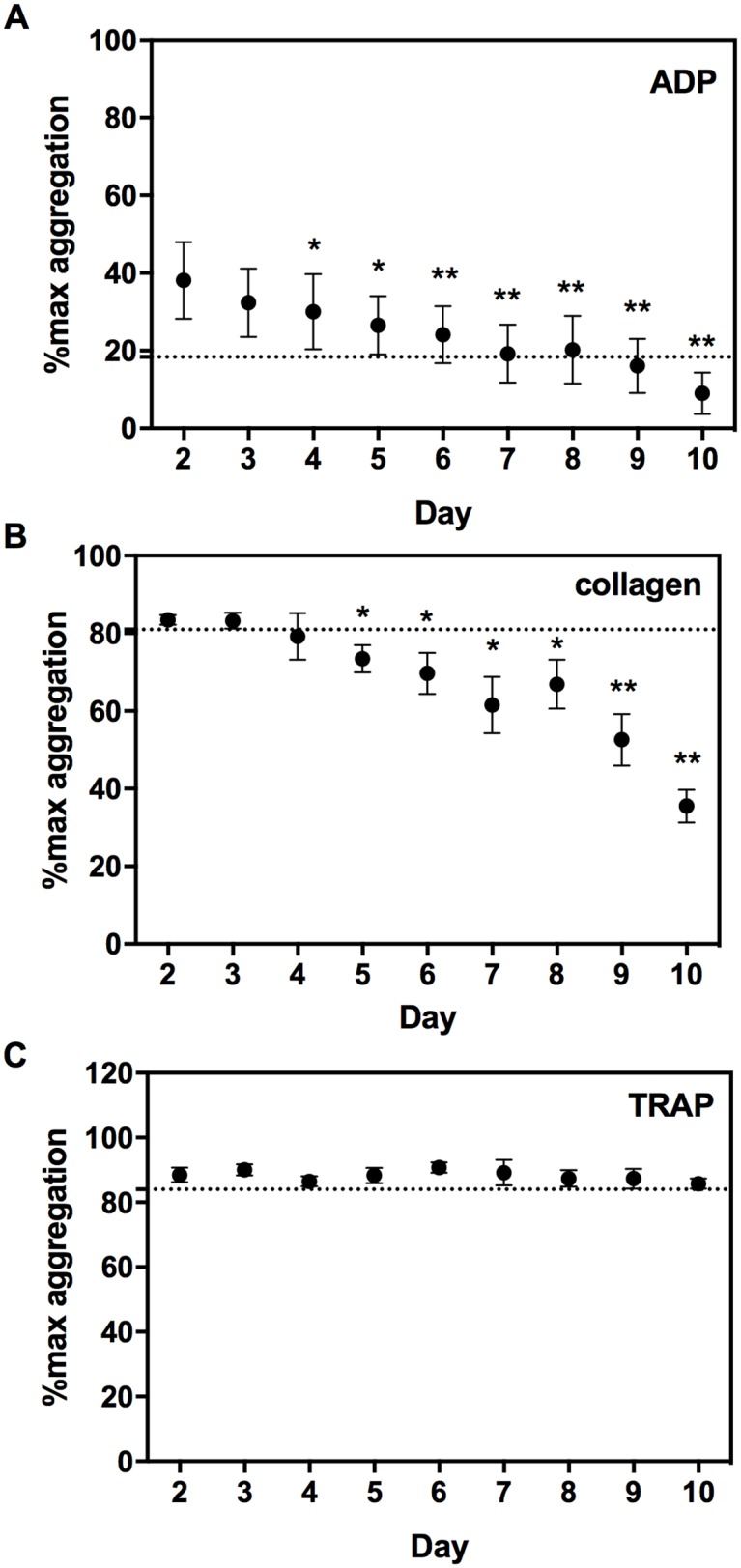
Effect of 10-day storage on platelet activation, measured by light transmission aggregometry (LTA). Aggregometry was measured in response to 10 μmol/L adenosine diphosphate (ADP, panel A), 4 μg/ml collagen (panel B) and 30 μmol/L thrombin receptor-activating peptide (TRAP-14, panel C). Light transmission in platelet concentrates (PCs) compared to autologous platelet poor plasma is expressed as percentage of maximal aggregation. Data are expressed as mean±SD and a dashed line was set on mean day 2—2SD. * = P<0.01, ** = P<0.001 compared to day 2.

LTA was performed to test the effect of storage on platelet function in the PCs. Each day, samples were taken out of the 5 PCs and platelets were activated with agonists ADP, collagen and TRAP-14 via the P2Y_12_, GPVI and PAR-1 receptors, respectively. Platelet aggregation triggered by ADP decreased in a linear fashion with -3.2% of maximum aggregation/day (*r*^*2*^ = 0.97; Fig 1A) and from day 9 on the mean dropped below 18% of the maximum aggregation (mean-2SD of day 2). Collagen-triggered aggregation decreased with -5.3% per day (*r*^*2*^ = 0.87; Fig 1B), and already at day 4 the mean of the maximum aggregation of the PCs dropped below 81% (mean-2SD of day 2). Aggregation induced by TRAP did not change significantly during 10 days of storage and the percentage of maximum aggregation remained above the mean-2SD of day 2 (Fig 1C).

### Optimized flow cytometric platelet activation test

To study the effect of storage on the reactivity of platelets, an optimized flow cytometric platelet activation test was used. Platelets were activated with the agonists ADP, CRP and TRAP-6 and dose-dependent activation graphs were created for P-selectin expression (see S1 Fig). Stimulation with 31 μmol/L ADP, 2500 ng/ml CRP and 10 μmol/L TRAP resulted in a maximal activation of the platelets and these concentrations were selected to study the effect of storage.

**Fig 2:**
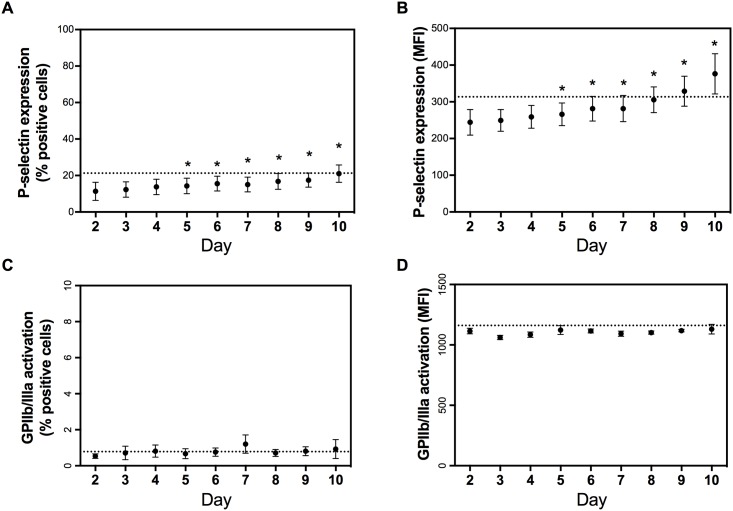
The effect of 10-day storage on spontaneous platelet activation by measuring P-selectin expression and activation of the GPIIb/IIIa receptor. The percentage of positive cells (panel A and C for P-selectin and activated GPIIb/IIIa, respectively) and the median fluorescence intensity on the platelets (panel B and D for P-selectin and activated GPIIb/IIIa, respectively) are shown. Data are expressed as mean±SD and a dashed line was set on mean day 2 +2SD. * = P<0.01 compared to day 2.

Storage of PCs had a moderate effect on spontaneous platelet activation (without agonist addition). After 2 days, already 11% of the platelets spontaneously expressed P-selectin on their outer surface and this percentage further increased in time up till 21% on day 10 after blood donation (>20%, mean+2SD of day 2; Fig 2 panel A). This spontaneous platelet activation was also reflected in the fluorescence measured on the cells (Fig 2 panel B). The P-selectin expression increased in time and significance was detected from day 5 after donation and from day 9 on, the mean P-selectin expression of the PCs was above 314 MFI (mean+2SD of day 2). Overall, the P-selectin expression increased linearly with 14.6 MFI/day (*r*^*2*^ = 0.88). In contrast, PCs storage had no significant effect on spontaneous activation of the GPIIb/IIIa receptor (Fig 2 panel C and D).

**Fig 3:**
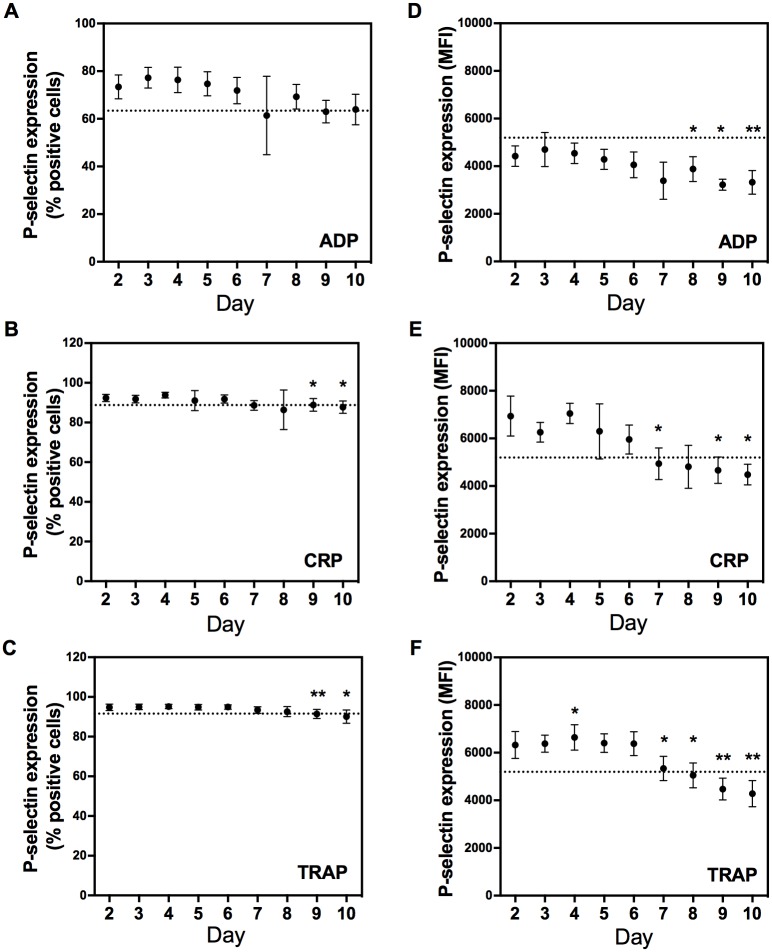
Effect of 10-day storage on platelet activation, measured by optimized flow-cytometric platelet activation test (PACT). Adenosine diphosphate (ADP, panel A and D), collagen-related peptide (CRP, panel B and E) and thrombin receptor-activating peptide (TRAP-6, panel C and F) respectively, were used to activate platelets. The percentage cells that express P-selectin (panel A-C) and the median fluorescence intensity (MFI) of the P-selectin positive cells (panel D-F) are shown. Data are expressed as mean±SD and a dashed line was set on mean day 2 -2SD. * = P<0.01, ** = P<0.001 compared to day 2.

The activation status of platelets was studied by measuring the percentage P-selectin positive cells after stimulation with agonists. At day 2, P-selectin expression was comparable in the different PCs and the mean percentage positive cells was 73%, 92% and 95% in response to ADP, CRP and TRAP, respectively. Storage of the PCs resulted in a significant linear decrease of the percentage P-selectin positive cells (-1.8%/day (*r*^*2*^ = 0.67), -0.7%/day (*r*^*2*^ = 0.68) and -0.6%/day (*r*^*2*^ = 0.80) after stimulation with ADP, CRP and TRAP, respectively; Fig 3A–3C). At day 7, 8 and 10 after donation, the mean percentage of P-selectin positive cells in response to CRP was lower than 89% (mean-2SD of day 2) and when PCs were activated with TRAP, the mean percentage of positive cells dropped below 92% (mean-2SD of day 2) at day 9 and 10 after donation. The mean percentage P-selectin positive cells in response to ADP never dropped below 63% (mean-2SD at day 2).

More important than the number of pre-activated platelets, is whether these cells have an adequate response when activated by agonists, measured by the amount of P-selectin expressed on the membrane of P-selectin positive platelets. Storage of the PCs resulted in a significant linear decrease over time of the amount of P-selectin expressed on the activated cells. When PCs were stimulated with ADP, CRP and TRAP, the fluorescence decreased with -184 MFI/day (*r*^*2*^ = 0.82), -341 MFI/day (*r*^*2*^ = 0.87) and -302 MFI/day (*r*^*2*^ = 0.81), respectively (Fig 3D–3F). The fluorescence dropped below 3562 MFI (mean-2SD of day 2) at day 7, 9 and 10 after donation, below 5261 MFI (mean-2SD of day 2) as of day 7 after donation and below 5195 MFI (mean-2SD of day 2) as of day 8 after donation when activated by ADP, CRP and TRAP, respectively.

## Discussion

Currently platelet function is not routinely evaluated to check the quality of PCs before administration to a patient, due to the lack of an efficient test [10]. Therefore, we tested if our optimized flow cytometry based PACT was able to detect decline of platelet function in platelet concentrates stored beyond both European and US storage guidelines (7 and 5 days after donation, respectively) [7, 8]. In agreement with LTA, the PACT showed that platelet function declined during storage of concentrates in response to platelet agonists. Of note, both tests have been optimized using different agonists, activating platelets via GPVI (collagen and CRP-XL for LTA and PACT, respectively) and PAR-1 (TRAP-14 and TRAP-6 for LTA and PACT, respectively) [11, 12, 14]. After careful consideration, it was chosen not to replace one with the other, because these agonists do not differ in their intrinsic activity. Storage of the PCs resulted in a significant increase of spontaneous P-selectin expression on the platelets, which is consistent with other recent work monitoring platelet function in apheresis concentrates using flow cytometry [16, 17]. In contrast, PCs storage had minimal effect on the activation of the GPIIb/IIIa receptor. This means that the increase in P-selectin expression on the platelet surface is due to a slow release of alpha granules and that storage of PCs does not result in a true activation of the platelets. A negative correlation between spontaneous P-selectin expression and *in vivo* recovery and survival time after platelet transfusion has been seen before and may be a relevant measurement in a clinical setting [18, 19]. Storage of PCs had a relatively small effect on the number of cells that become activated after stimulation with different agonists. However, the reactivity of the platelets to ADP, TRAP and CRP reduced significantly in time, corroborating previous work [14, 16, 17, 20].

In addition to the platelet function tests, we measured several metabolic parameters to monitor if our concentrates were stored adequately. Every effort was made to avoid bacterial contamination by using sterile equipment for sample collection, however, PCs were never tested for bacterial contamination. Platelets are metabolically active, which is reflected by a decrease of the pH and the glucose level, accompanied by an increasing lactate level, a finding consistent with previous work [18]. The pH decreased, however it remained above 6.8, the limit associated with disc-to-sphere shape transformation [15]. There were no relevant changes in platelet concentration, indicating that platelet number remained intact during the 10-day shelf time.

It is known for a few decades that flow cytometry is a reliable technique to quantify platelet activation with the expression of granule markers on the outer membrane of the platelets and with the activation GPIIb/IIIa on the platelet membrane [5]. We have made a stable reaction mix that can be stored at -20°C [14]. Furthermore, the fixated samples can be stored for up to a week at 4°C to allow analysis at a more convenient moment. The flow cytometric method described requires minimal labor, takes only 30 minutes to measure all 5 PCs together, and the technique itself has a good reproducibility, irrespective of operator experience [5]. Currently, processing and interpreting flow cytometric data remains a specialty skill, however in the near future computerized algorithms will subside the need for trained personnel.

The optimized PACT has several advantages over aggregometry. It needs only minimal volumes of PC (<50 μl concentrate) to test different platelet reactivity pathways, whereas LTA needs at least 2 ml of PC to produce platelet poor plasma and to perform the test with several agonists. LTA only allows measurement of as many samples or different agonists as the number of channels available, whereas flow cytometry can be automated to measure a large sample load. Additionally, LTA needs highly skilled staff to ensure reliable results [4]. Ideally, post-transfusion platelet function would be monitored using the same test, but most patients requiring PC transfusion suffer from thrombocytopenia. Aggregometry is known to be unreliable in case of extremes in platelet number [1, 3], and is therefore unsuitable to compare pre- and posttransfusion platelet function in thrombocytopenic patients. The PACT has been tested successfully before in thrombocytopenic patients, and may be useful to measure post-transfusion platelet function in such a cohort [14, 21]. Additionally, there is a scarcity of data regarding the suitability of aggregometry for predicting in vivo viability of PCs by ex vivo aggregation [13]. In this in vitro pilot study, we have not studied whether PACT is a good prognosticator for in vivo function of PCs after transfusion, but the optimized PACT is at least suitable for testing platelet function in both PCs and thrombocytopenic patients. Even though the current limited sample size precludes the ability to assess potential failure rate of PACT, the advantages of the optimized PACT lead to a new opportunity for monitoring the quality of PCs outside a research laboratory.

In summary, the current study shows that our optimized flow cytometric PACT is a feasible method to measure platelet function in concentrates. The test shows agreement with LTA with regard to decline in platelet activation during storage of concentrates. A major advantage of our modified flow cytometric PACT is that it is superior to both LTA and conventional flow cytometry based platelet function tests with regard to labor, time and therefore cost efficiency. The optimized PACT is ready to be validated on a larger scale for clinical applications. Furthermore, a post-transfusion study in a cohort of thrombocytopenic patients receiving PC transfusion, comparing decline of platelet function over time in stored PCs to post-transfusion platelet recovery and in vivo function is the next step in validating the PACT. This study would make the correlation of findings with clinical efficacy possible, and ultimately, allow speculation on the implications for transfusion practice.

## Supporting information

S1 Fig Dose-dependent activation of 5 platelet concentrates (PCs) at day 2 after blood donation. In order to determine optimal agonist concentrations, PCs were activated with 8 concentrations of respectively adenosine diphosphate (ADP; 125, 31.25, 7.81, 1.95, 0.49, 0.12, 0.03 and 0 μM) collagen-related peptide (CRP; 2500, 625, 156.25, 39.06, 9.77, 2.44, 0.61 and 0 ng/ml) and thrombin receptor-activating peptide (TRAP; 30, 10, 3.33, 1.11, 0.37, 0.12, 0.04, 0 μM). Platelet activation was measured as P- selectin expression in median fluorescence intensity (MFI). Data are expressed as mean±SD. From these data, 31.25 μM ADP, 2500 ng/ml CRP and 9.77 μM TRAP were found to induce optimal platelet activation in the 5 platelet concentrates tested.
(PDF)

S1 Database Database PACT. All raw data underlying Table 1, Figs 1, 2 and 3 and Supporting information 1 are included in the database.
(XLSX)
