# Supplementary figures and images for: Application of an optimized flow cytometry-based quantification of Platelet Activation (PACT): Monitoring platelet activation in platelet concentrates
Source: PLoS One. 2017 Feb 16;12(2):e0172265. doi: 10.1371/journal.pone.0172265 (PMC5313179; doi:10.1371/journal.pone.0172265)

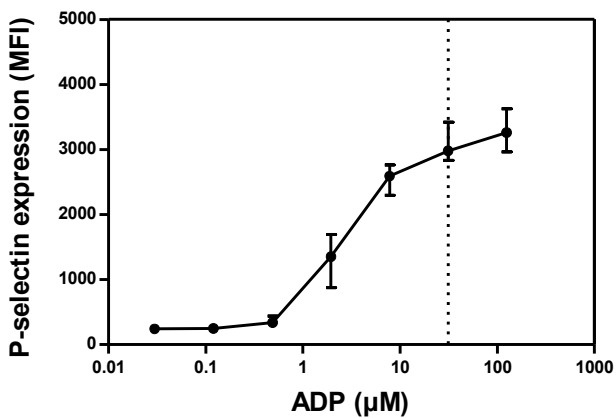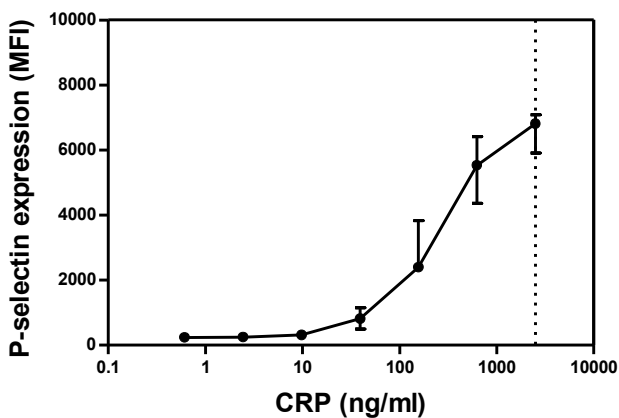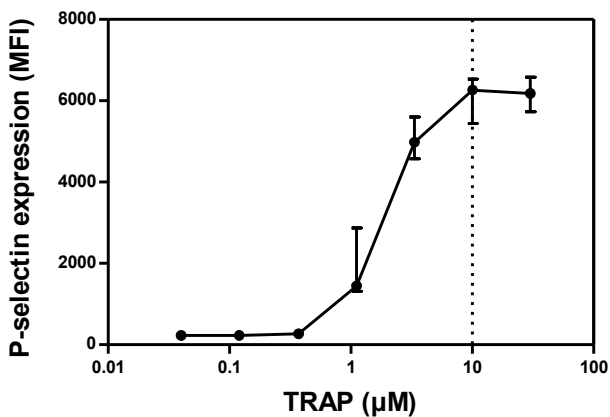

Supplement: S1 Fig — In order to determine optimal agonist concentrations, PCs were activated with 8 concentrations of respectively adenosine diphosphate (ADP; 125, 31.25, 7.81, 1.95, 0.49, 0.12, 0.03 and 0 μM) collagen-related peptide (CRP; 2500, 625, 156.25, 39.06, 9.77, 2.44, 0.61 and 0 ng/ml) and thrombin receptor-activating peptide (TRAP; 30, 10, 3.33, 1.11, 0.37, 0.12, 0.04, 0 μM). Platelet activation was measured as P- selectin expression in median fluorescence intensity (MFI). Data are expressed as mean±SD. From these data, 31.25 μM ADP, 2500 ng/ml CRP and 9.77 μM TRAP were found to induce optimal platelet activation in the 5 platelet concentrates tested. (PDF) [file pone.0172265.s001.pdf]
